# Social isolation recruits amygdala-medial prefrontal cortex projections to escalate alcohol drinking in male mice

**DOI:** 10.1101/2023.11.09.566421

**Published:** 2026-07-27

**Authors:** Reesha R. Patel, Kelly N. Kim, Makenzie Patarino, Rachelle Pamintuan, Felix H. Taschbach, Hao Li, Bitna Joo, Anna Pallé, Xianru Yu, Christopher R. Lee, Aniek van Hoek, Jesse White, Rogelio Castro, Christian Cazares, Raymundo L. Miranda, Caroline Jia, Jeremy Delahanty, Kanha Batra, Laurel R. Keyes, Avraham Libster, Romy Wichmann, Talmo D. Pereira, Marcus K. Benna, Kay M. Tye

## Abstract

Social isolation profoundly alters motivation and increases vulnerability to alcohol misuse in humans, yet the underlying neural mechanisms remain unclear. Here we show that isolation escalates alcohol drinking in male mice but suppresses it in females. Whole-cell recordings revealed that neurons in the basolateral amygdala projecting to the medial prefrontal cortex (BLA-mPFC) track alcohol intake in both sexes. Isolation increased BLA-mPFC excitability in males but decreased it in females, mirroring their opposite behavioral adaptations. Given this divergence, we focused subsequent mechanistic studies on males to isolate neural pathway-level drivers of escalated alcohol intake. Cellular-resolution calcium imaging showed that activity in BLA-mPFC neurons encodes and predicts alcohol drinking, and optogenetic activation of this pathway increased alcohol intake. Simultaneous optogenetics and calcium imaging revealed that BLA-mPFC stimulation enhanced mPFC neuronal responses to alcohol, mimicking isolation-induced activity patterns, while photoinhibition reduced drinking in isolated mice. Together, these findings identify a BLA-mPFC pathway mechanism through which social isolation reconfigures prefrontal processing to promote alcohol intake.

## Full Text Availability

The license terms selected by the author(s) for this preprint version do not permit archiving in PMC. The full text is available from the preprint server.

